# Airborne eDNA Reveals Resource‐Based Assembly of Frugivorous Vertebrates

**DOI:** 10.1111/1755-0998.70056

**Published:** 2025-10-10

**Authors:** Wang Cai, Yunao Li, Chunyan Yang, Zhaoli Ding, Wenfu Zhang, Viorel D. Popescu, Yang Jie

**Affiliations:** ^1^ Yunnan Key Laboratory of Forest Ecosystem Stability and Global Change, Xishuangbanna Tropical Botanical Garden Chinese Academy of Sciences Mengla Yunnan China; ^2^ School of Ecology and Environmental Sciences Yunnan University Kunming Yunnan China; ^3^ Genome Center of Biodiversity, Kunming Institute of Zoology Chinese Academy of Science Kunming Yunnan China; ^4^ Yunnan Key Laboratory of Biodiversity Information Kunming Yunnan China; ^5^ Yunnan International Joint Center of Urban Biodiversity Kunming Yunnan China; ^6^ Department of Ecology, Evolution and Environmental Biology Columbia University New York City New York USA; ^7^ Center for Environmental Studies University of Bucharest Bucharest Romania; ^8^ National Forest Ecosystem Research Station at Xishuangbanna Mengla Yunnan China

**Keywords:** biodiversity, environmental DNA, metabarcoding, tropical rainforest

## Abstract

Food availability is a fundamental driver of vertebrate spatial distributions, yet quantifying these relationships across taxonomic groups remains challenging in structurally complex ecosystems such as tropical rainforests. Understanding how resource heterogeneity shapes community structure is critical for advancing ecological theory and informing conservation strategies. We combined airborne environmental DNA (eDNA) sampling with ground‐based fruit tree surveys in the Xishuangbanna tropical rainforest to test whether measurable animal genetic traces in the air reflect the fine‐scale distribution of animals driven by fruit resources. Airborne eDNA sampling revealed a diverse vertebrate community, with 71 bird and 18 mammal species detected at high spatial resolution in tropical rainforests. By applying occupancy models to account for detection bias, we show that vertebrate occurrence patterns are significantly influenced by both the overall abundance of fruiting trees and, more importantly, the availability of small fruiting trees across locations. Our findings provide the first compelling airborne eDNA‐based evidence that fine‐scale fruit resource availability, particularly from small‐fruited trees, contributes to the spatial distribution of vertebrates in tropical rainforests of Xishuangbanna, China. These spatial couplings between plants and animals highlight the value of airborne eDNA not only for biodiversity monitoring but also for testing trait‐based ecological hypotheses (e.g., fruit size selection by frugivores) and advancing theory on community assembly in structurally complex ecosystems.

## 1 Introduction

Understanding ecological and evolutionary mechanisms that shape animal distribution has long been a central challenge in ecology. This challenge is particularly pronounced for highly mobile species, whose spatial patterns reflect a complex interplay of abiotic and biotic factors. At broad spatial scales, abiotic factors such as climate and topography are major drivers of species distributions (McGill 2010), while at finer scales, biotic factors, particularly food availability and interspecific interactions, become more influential (Hanya et al. 2011; Morellato et al. 2016).

Food availability is a central driver of animal movement and habitat use, as classically framed by optimal foraging theory (Pyke et al. 1977). This framework, now embedded within trait‐based models of consumer‐resource dynamics, predicts that animals optimize foraging efficiency by selecting areas offering the greatest energetic return (Mcgill et al. 2006). Empirical studies have consistently shown that frugivores tend to concentrate in areas of high fruit availability and expand their home ranges during periods of scarcity (Camaratta et al. 2017; Li et al. 2025). However, effective conservation requires strategies beyond increasing food availability; strategies must also incorporate how spatial configuration and trait‐based resource quality shape animal foraging strategies and habitat selection (Marshall and Wrangham 2007; Liu et al. 2025).

In tropical forests, fruit‐bearing trees provide a key food source for many birds and mammals (Gautier‐Hion et al. 1985), and their availability plays a central role in shaping the spatial and temporal distribution of frugivores (Rey 1995; Burns 2004). Empirical studies have documented strong relationships between fruit abundance and frugivore distribution at both local (e.g., within home ranges, Brown and Sherry 2008) and regional scales (e.g., migratory movements, Tellería and Pérez‐Tris 2003). Such patterns are consistent with predictions from trait‐based foraging frameworks, where animals select resources based on their own morphological and physiological constraints. Among morphological constraints, fruit size is a particularly important driver, as small‐bodied animals are generally unable to consume fruits exceeding their gape size (Guimarães et al. 2008; González‐Varo et al. 2022). These size‐based filtering patterns are thought to reflect long co‐evolution between frugivores and fruiting trees (Lim et al. 2020). However, the ongoing decline of large‐bodied frugivores may be altering these ecological and evolutionary relationships in ways that are not yet fully understood (Galetti et al. 2013). This is because large‐fruited trees are losing the large‐bodied frugivores that play a key role in their persistence through seed dispersal (Fricke et al. 2025). Therefore, understanding how fruit availability and traits shape frugivore distributions is essential for predicting forest dynamics and informing conservation strategies.

Fruit availability alone does not, however, always align with species distribution (Blendinger et al. 2012). Many frugivores have mixed diets that include insects, leaves, or nectar (Schleuning et al. 2011). Moreover, the degree of frugivorous specialisation may vary across space and time due to seasonal changes, geographic differences, and the diversity and abundance of tropical plants (Blüthgen et al. 2007; Daniel Kissling et al. 2009). These complexities challenge traditional approaches for mapping frugivore distributions. Methods such as foraging observations, mist nets, and camera trapping remain valuable, but are often taxonomically biased, spatially limited, and labour intensive (e.g., Saracco et al. 2004; Aristizabal et al. 2019). In the hyperdiverse tropical rainforest, where a single hectare can host over 650 woody plant species (Condit et al. 2005), uncovering spatial links between fruiting trees and frugivore communities requires extensive effort and deep taxonomic sampling (Seibold et al. 2018). Nonetheless, such cross‐taxonomic data are vital for understanding the functional role of frugivores in forest regeneration and broader ecosystem processes (Lundberg and Moberg 2003; Montoya et al. 2015).

To address the limitations of traditional surveys, we propose environmental DNA (eDNA) as a promising alternative to overcome challenges of traditional biodiversity sampling methods. Specifically, airborne eDNA has recently emerged as a novel approach for detecting terrestrial vertebrates across broad landscapes (Bohmann and Lynggaard 2023). By capturing genetic material suspended in the air, this approach enables detection of elusive or highly mobile species that are often missed by conventional methods (Garrett et al. 2023). Recent studies have shown that airborne eDNA can capture vertebrate signals under both controlled and natural conditions (Clare et al. 2022; Lynggaard et al. 2022, 2024). Unlike direct observation of foraging behaviour, airborne eDNA allows for broader spatial monitoring, as the airborne genetic signals are not restricted to particular trees or specific frugivores. Corroborating these findings, González‐Varo et al. (2014) successfully identified seed dispersers via DNA from seed surfaces, demonstrating that eDNA‐based detection of plant–animal interactions is feasible, though so far applied only to a limited number of species. However, due to the intuitive weakness and diffusion of airborne vertebrate eDNA signals, it remains unclear whether such data can reveal fine‐scale ecological associations, for which precise species distribution information is required, such as those shaped by fruit availability. Answering these questions would represent a critical step toward expanding airborne eDNA from a tool for species detection to one used for understanding ecological processes.

As natural ecosystems face rapid changes, bridging innovative tools with ecological research is increasingly necessary. Much of our current knowledge of frugivore–plant interactions relies on labor‐intensive field observations or species‐specific sample collection like faecal analysis (Jordano 1988; Schleuning et al. 2014). In this study, we integrate airborne eDNA sampling with a comprehensive tree census in a Forest Global Earth Observatory (ForestGEO) plot located in the Xishuangbanna tropical rainforest of southwest China to investigate how fruit availability shapes the spatial distribution of vertebrates. Specifically, we address two key questions motivated by optimal foraging theory and trait‐based ecology using airborne eDNA: (i) Can fine‐scale spatial distributions of vertebrates be predicted by fruit availability in the tropical rainforest ecosystem? We expect the richness of frugivorous species to be higher in areas with more fruiting trees. (ii) Does fruit size influence the spatial distribution patterns of vertebrate frugivores in this rainforest? We expect small species to have a higher probability of occupancy where there are more small fruits than large ones. By combining airborne eDNA sampling with eDNA‐aware occupancy modelling, we aim to establish a novel, interdisciplinary approach for understanding how consumer‐resource dynamics shape plant–animal interactions in complex tropical forest systems.

## 2 Materials and Methods

### 2.1 Study Site and Airborne eDNA Sampling

This study was conducted in a 20‐ha forest dynamics plot in Xishuangbanna, located within the Xishuangbanna National Nature Reserve in southwestern China. The plot is part of the ForestGEO global forest monitoring network (https://forestgeo.si.edu), which conducts a tree census every 5 years. Tree species richness is exceptionally high, making it one of the most diverse forest ecosystems in China. Species that produce fleshy fruits are common in the Xishuangbanna rainforest (66%), and small to medium‐sized fruits (< 20 mm in length) predominate (Chen et al. 2004). The study area is topographically heterogeneous, including three gullies with flat valley bottoms and steep surrounding slopes.

In November 2023, we deployed 20 airborne eDNA samplers across the 20‐ha forest dynamics plot, with one sampler per hectare to ensure maximum spatial coverage. The sampling time is not related to the peak of the fruiting period. No bait was used. The air samplers were modified from Garrett et al. (2023). Each sampler consisted of a 3D‐printed housing equipped with a detachable, sterilizable filter chamber designed to hold a disposable filter membrane. The internal components included a 4500 RPM motor, a rechargeable battery capable of operating the device for up to 30 h, and a fan connected to the motor used to filter air. Air was filtered at a rate of approximately 1 ± 0.3 m^3^/min using 47 mm diameter glass fibre filter membranes with a pore size of 0.45 μm. Sampling was conducted for three 24‐h occasions over 6 days, resulting in 60 eDNA samples (three replicates per sampling location). Before and after each sampling round, all equipment was decontaminated with 10% bleach and 75% ethanol, and accessories were sterilised in an autoclave. After sampling, each filter membrane was sealed in a sterile centrifuge tube, stored in ice‐packed coolers in the field, and immediately frozen at −20°C until DNA extraction. To monitor potential contamination, three field negative controls were included. Samplers were placed inside decontaminated, sealed plastic boxes (50 × 50 × 30 cm) and operated remotely for 6 h. These controls were handled identically to field samples during transport and storage.

### 2.2 Airborne eDNA Laboratory Processing

DNA was extracted using the DNeasy Blood & Tissue Kit (Qiagen GmbH, Germany). To reduce contamination risk, approximately 1 cm of the filter edge was removed. The remaining filter was cut into small pieces and placed into 2 mL tubes with 1.5 mL of digestion buffer and incubated at 56°C for 24 h. The remaining steps followed the manufacturer's protocol. Three DNA extraction negative controls were prepared by operating samplers in the extraction room for 6 h and then extracting DNA using the same protocol as field samples.

For metabarcoding, we targeted a 73–110 bp fragment of the 12S rRNA gene using the 12SV05 universal primer set for vertebrate amplification (Riaz et al. 2011). Mixed artificial DNA (0.05 ng/μL) from five species which are not found in the wild in China was used as a positive control to indicate successful PCR amplification. The five positive control species are *Ceratotherium simum* , *Rangifer tarandus* , *Diomedea exulans* , *Apteryx australis* , and *Triturus cristatus* . A 150 bp synthetic DNA sequence was inserted into a plasmid vector for each species. This DNA can be amplified using the 12SV05 primer. The synthetic DNA was produced by Sangon Biotech (Shanghai, China) Co. Ltd. To minimise the risk of high‐abundance species masking low‐abundance ones, we optimized PCR conditions (eDNA template concentration and PCR cycle number) using SYBR Green qPCR following the approach in Yang et al. (2021). Briefly, we conducted 50‐cycle qPCR with four DNA template concentrations (undiluted, 5×, 10×, and 25× dilutions). The concentration showing the earliest amplification onset and still amplification to the end was selected as optimal. The optimal cycle number was set at the minimum cycle within the amplification plateau phase, since amplification beyond this phase does not increase diversity but may allow high‐abundance species to mask low‐abundance ones (Murray et al. 2015). Based on qPCR results, we selected 2 μL of undiluted template and 40 cycles for optimal amplification. Thus, each 20 μL PCR reaction contained 2 μL eDNA template, 10 μL 2× AmpliTaq Gold Master Mix (Applied Biosystems), 2 μL mixed tagged forward and reverse primers (10 μM), and 6 μL double distilled water. PCR conditions included: initial denaturation at 95°C for 10 min; 40 cycles at 95°C for 30 s, 57°C for 30 s, 72°C for 30 s; and final extension at 72°C for 5 min. Three PCR negative controls were included by adding 2 μL of double‐distilled water instead of eDNA template.

**FIGURE 1:**
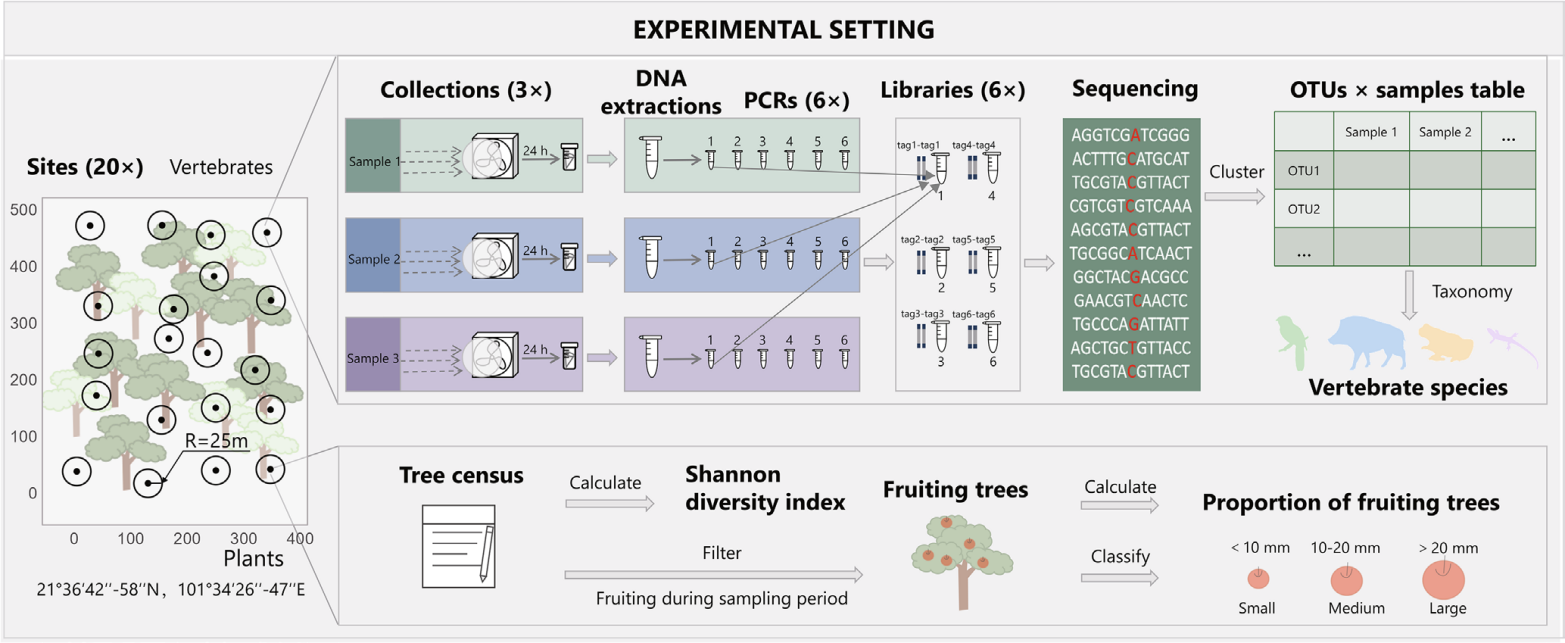
Overview of the study design and sampling workflow. Airborne eDNA samplers were deployed to detect vertebrate presence at each location (black dot), while fruiting tree data were collected through systematic field surveys and phenological monitoring. For each eDNA sampling location, fruit tree data were extracted within a 25‐m radius (black circle). The integration of these datasets enables the examination of spatial coupling between animal occurrence and fruit availability.

Each sample was amplified in six independent PCR reactions using unique 8–9 bp twin‐tagged primer pairs, allowing replicate tracking during downstream bioinformatics and modelling. In twin‐tagging, the same tag sequence is added to both forward and reverse primers (e.g., F1–R1, F2–R2). For each sample, a different twin tag was used for each of the six PCR replicates, which were pooled into six separate libraries (e.g., sample1–F1–R1 in library 1, sample1–F2–R2 in library 2, …). This strategy ensured unambiguous identification of PCR replicates in bioinformatic processing. PCR products then were visualised using 2% agarose gel electrophoresis and quantified with a Qubit 2 fluorometer (Thermo Fisher Scientific). All 414 PCR products (6 per sample × 60 samples, and 6 per control × 9 controls) were pooled into six roughly equimolar libraries, each containing one PCR product per sample (Figure 1). Samples with low DNA yield (below Qubit quantify threshold) were pooled using 10 μL of product. Final pools were purified using KAPA magnetic beads (ratio 1:1), and six libraries were built with the NEXTflex Rapid DNA‐Seq Kit (Bioo Scientific). Sequencing was performed on the Illumina NovaSeq 6000, using NovaSeq 6000 SP Reagent Kit v1.5 (300 cycles). All lab work was conducted at the Kunming Institute of Zoology, China.

### 2.3 Bioinformatic Processing and Taxonomic Assignment

We obtained a total of 337,285,688 paired‐end sequences from six sequencing libraries, averaging 4,888,194 sequences per sample. Raw reads were quality‐trimmed to remove adapter sequences and low‐quality bases (QC < 30, short reads, or reads with excessive Ns) and were merged (paired reads) using fastp v0.20.1 (Chen et al. 2018). Merged paired reads were then demultiplexed and filtered for tag‐jumping using the Begum pipeline (An updated version of DAMe, Zepeda‐Mendoza et al. 2016). We allowed primer identification up to two mismatches and only sequences with at least 10 copies and lengths between 80 and 120 bp were retained. Unlike the original design of the Begum pipeline, which filters sequences based on their occurrence across multiple PCR replicates to minimize sequencing errors, we retained sequences from each individual PCR replicate for each sample. This approach allows us to incorporate detection probabilities in the downstream occupancy modeling, as described below. Chimeric sequences were subsequently removed using the de novo chimera detection function in VSEARCH v2.28.1 (Rognes et al. 2016). Sequences were clustered into OTUs (Operational Taxonomic Units) at 97% similarity using sumaclust v1.0.31 (Mercier et al. 2013), resulting in an OTU‐by‐sample matrix.

Taxonomy was assigned to OTU representative sequences using BLAST (blastn) against the NCBI GenBank nucleotide database (version 20240208). Assignments required ≥ 99% identity across the entire query and an E‐value < 1e‐5. If multiple 100% matched species occurred, we assigned the OTU to the species known to distribute in the study area. Where multiple 100% species matches were found and they are locally distributed, the OTU was assigned to the lowest common taxonomic level shared by multiple 100% matched species to ensure accuracy and to remove ambiguous assignments (e.g., assigned to genus level). Species distributions were checked with the Global Biodiversity Information Facility (GBIF) and regional published research (see SM1).

OTUs assigned to humans and domestic animals (e.g., pigs, chickens, cows, sheep, dogs, and cats) were removed, as these are common sources of eDNA contamination. This could be due to humans carrying them, garbage in the environment, and human food. Human, pig, and chicken DNA also appeared in field negative controls. Although the assignment of pig and chicken could be wild boar and red jungle fowl, which are wild sources, we could not distinguish them from domestic animals using the 12SV5 primer. Thus, the following analysis excludes wild boar and red jungle fowl. After excluding sequences from domestic species and humans, the negative controls showed no evidence of vertebrate taxa. Amphibians, reptiles, and fish were also excluded, as we assumed their presence is unrelated to fruit traits.

After quality control above, we retained 373,986 reads representing 89 terrestrial vertebrate species across the 60 wild samples. Species traits (diet category and body mass) were obtained from EltonTraits 1.0 (Wilman et al. 2014). We classified frugivores as species whose diets contain more than 10% fruit or seed, and non‐frugivores as species whose diets contain no fruit or seed. Non‐frugivores were further divided into carnivores, defined as species consuming only animal matter, and omnivores, defined as species whose diets include both plant material (excluding fruit or seed) and animal matter. The details of body mass, diet, IUCN conservation status, and diet category used in this study for each species are provided in Table S1.

### 2.4 Environmental Covariates

We used seven environmental covariates representing topography and forest structure to model occupancy and species‐environment relationships. Topographic variables included mean elevation and mean slope within a 25‐m radius of each sampling location. Forest structure variables included canopy closure, Shannon diversity index of tree species, and the proportion of fruiting trees (or by fruit size class) within a 25‐m radius of each sampling location. All data (except for canopy closure, which is measured during sampling) were derived from the 2022 forest plot monitoring conducted by the Xishuangbanna Tropical Botanical Garden, measured using the ForestGEO standard (Davies et al. 2021).

The tree inventory used to obtain the forest structure variables included all living trees with diameter at breast height (DBH) ≥ 1 cm. Fruit period and size were extracted from the Flora of China (http://www.iplant.cn). Of the 289 species with fruiting period and fruit size data (covering 71% of the plot's species), 125 were potentially fruiting during the sampling period. We calculated the proportion of fruiting trees to all trees within a 25‐m radius of each sampling location. Then, fruiting trees were grouped into three fruit size classes: small (< 10 mm), medium (10–20 mm), and large (> 20 mm), according to Dong et al. (2023). All covariates were standardised (mean = 0, SD = 1) using the scale() function in R.

### 2.5 Occupancy Model

We used *occupancyPlus* (Ji et al. 2025), a variant of the hierarchical Bayesian multi‐species occupancy model *eDNAPlus* (Diana et al. 2024), which was specifically developed for eDNA data. Wild animal surveys often face the problem of imperfect detection (false negatives), which can lead to biased estimates of species distributions (MacKenzie et al. 2002; Tyre et al. 2003). To address this issue, occupancy models have been widely used to correct for imperfect detection in traditional survey methods such as camera traps (Doser et al. 2022; Rozylowicz et al. 2024). However, these models cannot be directly applied to eDNA data because the sources of detection error in eDNA studies differ from those in camera trapping (Burian et al. 2021). For instance, camera traps typically involve continuous monitoring at a single site over an extended period, and the models are designed to correct for detection probability over time. In contrast, eDNA sampling usually involves multiple replicate collections over a short period at each site and therefore requires models that account for replicate‐based sampling error. In addition, eDNA metabarcoding data are affected by false positive and negative errors during laboratory processing, such as cross‐sample contamination and random loss of rare sequences during PCR amplification (Cristescu and Hebert 2018), respectively. For these reasons, occupancy models tailored to eDNA data have been recently developed (e.g., Fukaya et al. 2021; Diana et al. 2024; Ji et al. 2025).

In occupancyPlus, detection is modelled at two stages. The first stage models the probability of capturing species DNA in an air sample, given presence at the site, accounting for sampling replicates. The second stage models the probability of detecting DNA in PCR (here, one extraction for one field sample), conditional on its presence in the sample, accounting for PCR replicates and read counts. The model also accounts for false positives, which is also a common problem in eDNA studies (Ficetola et al. 2015). For modelling occupancy probability of individual species, the model supports an N‐dimensional latent factor approach, which allows us to efficiently display sites that are similar to each other with low dimension. Inference in OccPlus is performed using the software Stan v2.36.0 (Team 2024), which uses Variational Bayes to achieve fast inference. We fitted models with 1–3 latent factors for each group and compared predictive performance using leave‐one‐out cross‐validation (LOO). The model with the highest expected log predictive density (ELPD) was selected to ensure an adequate representation of the latent factor structure. We selected one‐ and two‐dimensional latent factors (*d* = 1 or 2) for the diet classification and taxonomic group analyses, respectively. For more model details, see Ji et al. (2025) and Diana et al. (2024).

We assumed species detection and occupancy probabilities varied by species and location characteristics. For detection probabilities, all locations followed the same sampling protocol (same sampling time, wind amount, preservation, and extraction protocol), so no location‐specific detection covariates were included in the model. For occupancy probabilities, we modelled occupancy using slope and elevation (topography) and canopy closure, tree diversity (Shanon diversity index), and fruiting tree or fruit size proportion (forest structure). We used the posterior means and 95% Bayesian credible intervals to assess the significance of covariate effects. The effect of a covariate on occupancy was considered significant if its 95% CI did not overlap zero. For model fitting, species detected at only one location were excluded due to low detectability and limited contribution to model stability, leaving 65 species (14 mammals and 51 birds) for analysis. We modelled mammals and birds separately and separated frugivores and non‐frugivores due to ecological differences. All model results were visualised using the R package ggplot2 v3.5.2 (Wickham 2016).

We show the conceptual framework that integrates airborne eDNA sampling with fruiting tree surveys to assess fine‐scale ecological associations between vertebrate communities and fruit resources in a tropical rainforest in Figure 1. This figure was created using Microsoft PowerPoint 2021.

## 3 Result

**FIGURE 2:**
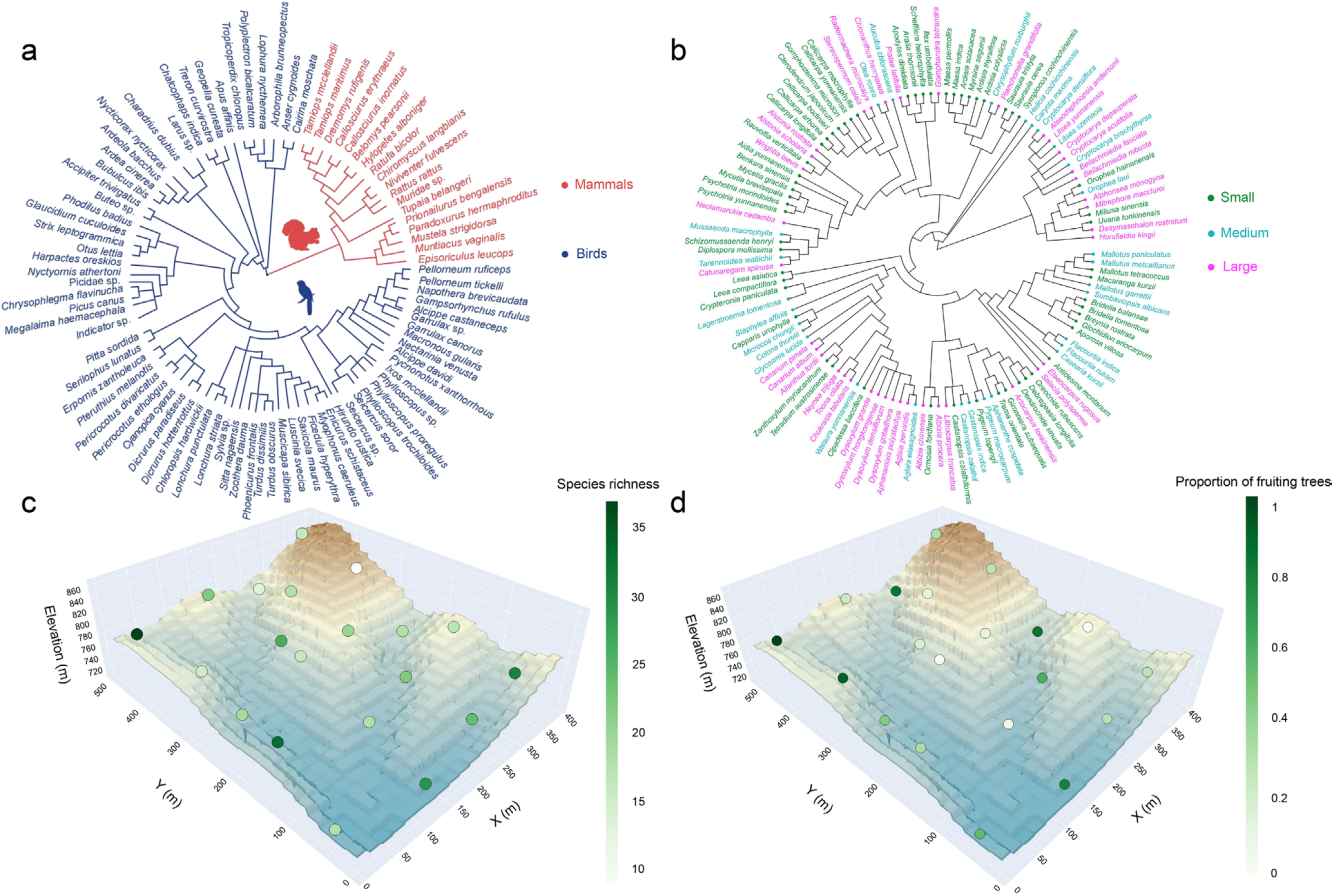
Species composition and richness of vertebrates and fruiting plants across sampling locations. Phylogenetic trees showing the taxonomic composition of terrestrial vertebrates detected by airborne eDNA (a) and fruiting plants during the sampling period across all locations (b). Three‐dimensional map showing the topographic structure of the study plot, with (c) showing the vertebrate species richness detected at each sampling location, and (d) showing the relative abundance of fruiting plants across sampling locations within a 25 m radius.

A total of 89 terrestrial species were detected using airborne eDNA, including 71 bird species and 18 mammal species (Figure 2a). The detection of location‐level vertebrate richness is shown in Figure 2c. Out of 289 fruit‐bearing tree species, 125 species were identified as potentially fruiting during the sampling period (Figure 2b,d).

**FIGURE 3:**
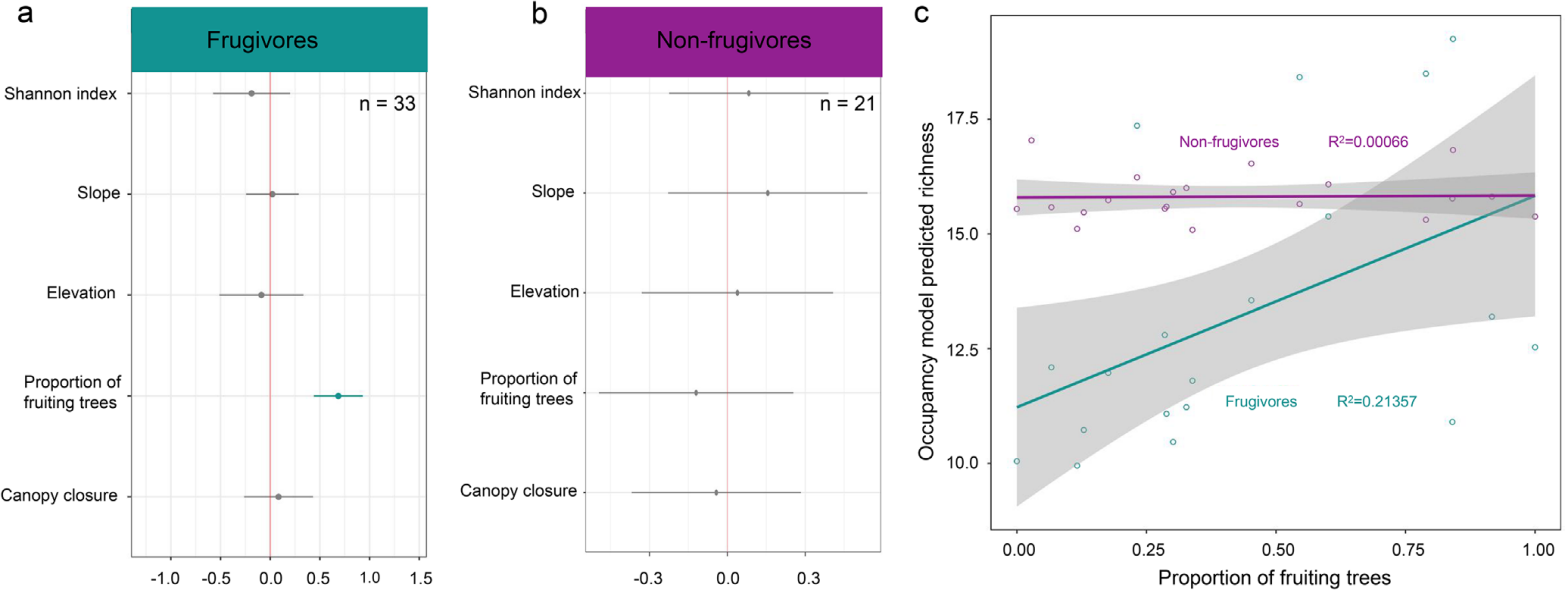
Estimated site occupancy coefficients and 95% Bayesian credible intervals (95% CI) for (a) Frugivores and (b) Non‐frugivores: Each bar represents the effect size of a specific coefficient on site occupancy. The green bar indicates variables with statistically significant effects (credible intervals not overlapping zero). The number of species included in the analysis is indicated by the letter ‘*n*’. (c) The relationship between the model predicted species richness (the sum of the species‐level occupancy probabilities for each location) and the proportion of fruiting trees at each location. Lines show the generalised linear model fitting for frugivores (green line) and non‐frugivores (purple line), respectively, with shaded areas representing 95% confidence intervals.

Occupancy modelling incorporating vertebrate detections, topographic features, and forest structure variables revealed that the proportion of fruiting trees was a significant predictor of frugivore presence (Figure 3a, *p* < 0.05). Model predicted frugivore richness was positively associated with the proportion of fruiting trees at each location (Figure 3c, *p* < 0.05, *R* ^2^_GLM_ = 0.21). In contrast, the proportion of fruiting trees had no significant impact on the occupancy of non‐frugivores (Figure 3b,c), and this pattern was consistent for both carnivores and omnivores (Figure S2). Topographic variables such as elevation and slope, as well as vegetation structure metrics—including overall plant diversity (Shannon index) and canopy closure—did not significantly explain variation in frugivore occupancy. Among these, slope showed importance for mammal richness, though its influence was weaker than that of fruit resource availability (Figure S1, *p* < 0.05).

**FIGURE 4:**
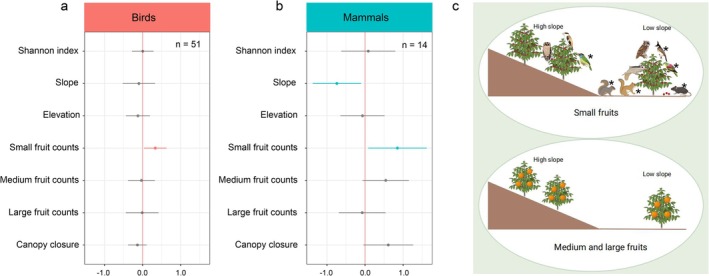
Estimated site occupancy coefficients and 95% Bayesian credible intervals (95% CI) for (a) birds and (b) mammals. (a) Effect sizes of covariates on occupancy probabilities for birds; (b) effect sizes for mammals. Each bar represents the estimated effect of a covariate on site occupancy, with red or blue bars indicating statistically significant effects (95% credible intervals do not overlap zero). The number of species included in the analysis is indicated by the letter ‘*n*’. (c) Representation of habitat preferences, summarising the results from (a) and (b). The top panel shows that mammals prefer flatter areas with higher densities of small‐fruited trees, whereas birds are associated with small‐fruited trees regardless of slope. The bottom panel indicates that habitats dominated by medium‐ to large‐fruited trees are not preferred by either mammals or birds. Species marked with an asterisk (*) show significant occupancy responses to the corresponding covariates (i.e., both birds and mammals respond significantly to small‐fruited trees, and mammals also respond significantly to slope).

We then tested whether the distribution of birds and mammals was affected by topographic variables and fruit size. The analysis revealed a consistent pattern of fruit size preference among both birds and mammals (Figure 4, Figure S3). Both groups showed higher occupancy probabilities in areas dominated by small‐fruited tree species (Figure 4a,b, *p* < 0.05), with little to no association with medium‐ or large‐fruited species. Additionally, mammals exhibited a preference for lower slope areas (Figure 4b, *p* < 0.05), whereas birds showed no significant response to topographic variables within this fine‐scale plot (Figure 4a). Furthermore, three mammal species showed the highest occupancy in areas with small‐fruiting trees: one Muridae species and two squirrel species ( *Dremomys rufigenis* and *Tamiops maritimus* ) (see Figure 4c, species occupancy was found to be significantly associated with small fruit size, *p* < 0.05). Similarly, several frugivorous bird species, such as the highly frugivorous *Megalaima haemacephala* , *Treron curvirostra* , and *Pycnonotus jocosus* , also favoured areas with a higher density of small‐fruiting trees.

## 4 Discussion

By integrating airborne eDNA with tree inventory data in a standardized forest dynamic plot in the ForestGEO network, we reveal that frugivorous birds and mammals are more likely to occupy areas with higher proportions of fruiting trees, whereas non‐frugivorous species show no such pattern in the tropical rainforest. These findings support the ecological expectation that fruit availability is a key driver of frugivore distribution at the community level, and the body size matches the fruit size that they prefer, now detected across multiple taxa through airborne eDNA. These findings provide empirical airborne eDNA‐based demonstrations of spatial coupling between fruit resource availability and vertebrate distribution in tropical forests. More broadly, they showcase the potential of airborne eDNA for efficiently uncovering fine‐scale ecological processes across taxonomic groups that have been difficult to access using traditional approaches.

### 4.1 Fruit Resource Predicted Vertebrate Occupancy

Fruit is a key food resource for many tropical animal species. Mutualistic networks between frugivores and plants suggest that species whose diets include fruits and seeds are not randomly distributed, but are structured by ecological interactions and resource availability (Jordano 2000; Bascompte and Jordano 2007). Therefore, the spatial and temporal dynamics of frugivores should, in principle, be predicted by fruit availability. This association has been supported by previous studies: in the Amazon, for example, positive relationships were observed between the abundance of certain tanager species and local fruit availability (Saracco et al. 2004). In the subtropical Andean mountain forests, frugivore abundance tracked fruit availability based on observational records of fruit‐eating birds (Blendinger et al. 2012). These findings collectively suggest that frugivores are accurately tracking fruit resource availability across different tropical ecosystems.

Our findings support and extend these observations, demonstrating that fruit availability significantly predicts frugivore spatial distribution in a tropical rainforest in southwest China. During the resource‐scarce dry sampling season, fruit availability was an important driver of frugivore occupancy at a fine spatial scale (< 20 ha), affecting both birds and mammals (Figure 3, Figure S1). This suggests a general pattern across multiple taxa. In total, we identified 23 bird species and 12 mammal species that are resident in the study area and rely partially or primarily on fruit as a food source, representing a large proportion of the frugivore genera known from the region. Some species, such as Muridae species and two squirrels ( *Dremomys rufigenis* and *Tamiops maritimus* ), exhibited particularly strong responses to fruit abundance. These species have previously been identified as key frugivores and seed dispersers in this region (Tongkok et al. 2020), and our findings reinforce their ecological importance. Additionally, mammal occupancy was positively associated with flatter terrain. This pattern may be due to untested indirect environmental factors or species interactions (e.g., flatter areas offering more prey and higher hunting success). In contrast, birds, due to their higher mobility, were less restricted by topography. Together, these results highlight the combined roles of food and topography in shaping mammal distributions.

While many species contribute to frugivore–plant interaction networks, only a subset appears to play a disproportionate role in maintaining the structure and function of these networks (Ramos‐Robles et al. 2018). In addition, closely related species may exhibit divergent foraging behaviours and fruit‐tracking strategies. For example, in the Amazon, different primate species showed distinct responses to fruit availability, with some congregating around specific fruiting trees (Mourthé 2014). In our study, we detected six squirrel species, but only two responded strongly to fruit resource availability. Given the regional decline of large‐bodied frugivores, squirrels have emerged as major fruit consumers and seed dispersers throughout southwest China's tropical forests (Naish 2015; Estrada et al. 2017; Menon and Tiwari 2019). However, our data were collected over only 6 days within a single season at a fine scale; the dataset is insufficient for a robust ecological network analysis that could clarify the role of each species within the frugivore–plant interaction networks. Further research incorporating species‐specific and temporal variation will be necessary to better predict long‐term forest dynamics.

### 4.2 Fruit Size Selection by Small Frugivores

Beyond the overall proportion of fruiting trees, we found that both birds and mammals exhibited higher occupancy in areas dominated by small‐fruited tree species (Figure 4). This pattern is likely explained by the frugivore community composition in our study area, which is dominated by small‐bodied species (body mass < 5000 g). These results add to growing evidence that fruit size—not just quantity—acts as an ecological filter influencing frugivore spatial behaviour and resource use (e.g., Jordano 2000; Lim et al. 2020). Specifically, small‐bodied animals are limited by gape size and are more likely to consume small fruits. Such fruit–frugivore size matching reflects long‐term coevolutionary processes and has been documented in systems such as palm–frugivore interactions (Lim et al. 2020).

Large‐bodied frugivores were largely undetected in our study. Some regionally distributed species, such as *Macaca mulatta* , *Tragulus kanchil* , and *Macaca leonina* , are rare or infrequent visitors to the study area, while others (e.g., *Muntiacus muntjak* ) were excluded from analysis due to low detection rates. Consequently, we could not assess their fruit size preferences and selection. Nevertheless, the absence or rarity of large frugivores in our sampling highlights a broader concern: the ongoing decline of large‐bodied frugivores (e.g., hornbills, ungulates, primates, elephants) may change long‐standing fruit–frugivore coevolutionary dynamics (Galetti et al. 2013).

More than 80% of woody plant species in tropical rainforests produce fleshy fruits that depend on animals for seed dispersal—an essential process for regeneration and maintaining plant diversity (Jordano 2000). In our study area, 221 tree species produce medium to large fruits (> 20 mm). Without large frugivores to disperse them, these trees may experience reduced regeneration success. Studies in defaunated Amazonian forests suggest that large fruits may evolve toward smaller sizes to adapt to dispersal by smaller animals (Galetti et al. 2013), while others may suffer recruitment failure and edge toward extinction if suitable dispersers are no longer present (Brodie 2017). This situation underscores the urgent need for frugivore conservation in southwest China and across South Asia. Large‐bodied frugivores such as hornbills, primates, and elephants are not yet extinct in the region but are increasingly restricted in range and declining in abundance (Naish 2015; Estrada et al. 2017; Menon and Tiwari 2019). Protecting these species not only conserves animal diversity but also safeguards plant diversity by enabling seed dispersal across larger spatial scales (Lamperty and Brosi 2022). Even where large‐fruited species persist, remaining large frugivores may be unable to access them due to habitat fragmentation. Promoting transboundary conservation efforts can help reconnect these mutualistic networks—facilitating both the movement of large frugivores and the regeneration of plant species that rely on them. Such actions are critical for reversing biodiversity loss in the face of escalating human pressures and climate change (Fricke et al. 2025).

### 4.3 Advancing Ecological Pattern and Process With Airborne eDNA

Environmental DNA (eDNA) has rapidly evolved into a scalable and effective tool for monitoring biodiversity across large spatial scales (Cristescu 2014). While aquatic eDNA is now widely applied to ecological questions ranging from single‐species detection to community‐level inference (Goldberg et al. 2016), airborne eDNA is emerging as a powerful approach for terrestrial systems. Initially used to monitor airborne microorganisms such as bacteria, fungi, and pollen (Johnson et al. 2021; Sánchez‐Parra et al. 2021), recent work has shown that it can also capture genetic material from elusive terrestrial vertebrates (Bohmann and Lynggaard 2023). This breakthrough extends biodiversity monitoring into forest canopies and remote, dry habitats that were previously inaccessible using aquatic eDNA (Kirchgeorg et al. 2024). Nevertheless, airborne eDNA shares many of the inherent limitations of other eDNA approaches. Challenges include potential inaccuracies in species identification and false negatives, which may arise from primer bias, bioinformatic processing, or incomplete reference databases (Cristescu and Hebert 2018). In addition, rigorous experimental design is essential to minimize contamination (Blackman et al. 2024). A further challenge to airborne eDNA is the typically low concentration of vertebrate DNA in the air, which complicates cost‐efficient collection and storage (Lynggaard et al. 2024). Together, these limitations currently constrain the reliability and efficiency of airborne eDNA for vertebrate species detection.

Yet the central question is not only whether species can be detected, but whether airborne eDNA can be used to investigate ecological processes, such as species interactions and resource tracking, across spatial and temporal scales. Our study shows that integrating high‐resolution airborne eDNA sampling of vertebrates with fruiting tree distributions in a tropical rainforest can reveal how fruit resources influence vertebrate community patterns. Importantly, these findings, derived from a short sampling period, suggest that airborne eDNA captures fine‐scale, real‐time ecological drivers of animal presence rather than simply reflecting background biodiversity signals. However, the transport dynamics of airborne eDNA remain a major limitation when it comes to interpreting spatial and temporal patterns. While Tournayre et al. (2025) reported that airborne eDNA can travel over long distances, Bodawatta et al. (2025) suggested that detectable signals rarely disperse far due to dilution and degradation. The environment in which the study is conducted may also play a role: airborne eDNA could travel further in open fields than in the understorey of a dense forest. Resolving this uncertainty will require distance–decay experiments to improve our understanding of the spatial resolution of airborne eDNA.

Overall, beyond technical validation, our findings carry broader ecological implications. Fruit availability, a central axis of consumer‐resource interactions in tropical ecosystems, emerged here as a predictor of the spatially distributed structure of vertebrates—detectable purely from airborne eDNA. This reinforces the idea that food resource filtering is not only a theoretical concept but one that leaves measurable genetic footprints in the air. As airborne eDNA is deployed across broader spatial and temporal gradients, it may offer new insights into how trophic interactions shift with climate change, habitat fragmentation, or faunal loss (Seibold et al. 2018). Moreover, combining airborne eDNA with advanced modelling frameworks—such as occupancy‐based joint species distribution models—and long‐term monitoring platforms like ForestGEO presents a powerful opportunity to explore how functional traits and mutualisms shape the spatial organisation of biodiversity (Rumeu et al. 2020; Cai et al. 2025).

## Author Contributions

Wang Cai conceived the ideas; Wang Cai, Yunao Li, and Wenfu Zhang collected the data; Wang Cai, Viorel Popescu, and Yunao Li analyzed the data; Wang Cai led the writing of the manuscript. All authors contributed critically to the drafts and gave final approval for publication.

## Conflicts of Interest

The authors declare no conflicts of interest.

## Supporting information


**Figure S1:** Estimated site occupancy coefficients and 95% Bayesian credible intervals (95% CI) for (a) birds and (b) mammals. (a) Effect sizes of covariates on occupancy probabilities for birds; (b) effect sizes for mammals. Each bar in the graph shows the estimated effect of a covariate on site occupancy. Bars indicate statistically significant effects, where the 95% credible intervals do not overlap zero.
**Figure S2:** Estimated site occupancy coefficients and 95% Bayesian credible intervals (95% CI) for (a) Frugivores and (b) Carnivores (c) Omnivores: Each bar represents the effect size of a specific coefficient on site occupancy. The green bar indicates variables with statistically significant effects (credible intervals not overlapping zero). The number of species included in the analysis is indicated by the letter ‘*n*’.
**Figure S3:** The influence of fruit size classes on the occupancy of single species. The colour indicates the strength of the effect of fruit size. We fitted a beta regression model for each species and extracted standardised coefficients for each fruit size.

**Table S1:** This table lists the taxonomic information of all species detected by airborne eDNA sampling alongside their body mass, diet, IUCN conservation status and diet category, as defined in this study. ‘Low detection’ in the classification means that the species was detected at fewer than two sites and was therefore not included in the analysis.

## Data Availability

Sequence data are under GenBank accession number PRJNA1265915. The OTU table and codes for the occupancy model can be found on https://doi.org/10.5281/zenodo.17096327.
