# Supplementary material for: Airborne eDNA Reveals Resource‐Based Assembly of Frugivorous Vertebrates
Source: Mol Ecol Resour. 2025 Oct 10;25(8):e70056. doi: 10.1111/1755-0998.70056 (PMC12550463; doi:10.1111/1755-0998.70056)
Supplement: Supplementary file 1 — Figure S1: Estimated site occupancy coefficients and 95% Bayesian credible intervals (95% CI) for (a) birds and (b) mammals. (a) Effect sizes of covariates on occupancy probabilities for birds; (b) effect sizes for mammals. Each bar in the graph shows the estimated effect of a covariate on site occupancy. Bars indicate statistically significant effects, where the 95% credible intervals do not overlap zero. Figure S2: Estimated site occupancy coefficients and 95% Bayesian credible intervals (95% CI) for (a) Frugivores and (b) Carnivores (c) Omnivores: Each bar represents the effect size of a specific coefficient on site occupancy. The green bar indicates variables with statistically significant effects (credible intervals not overlapping zero). The number of species included in the analysis is indicated by the letter ‘n’. Figure S3: The influence of fruit size classes on the occupancy of single species. The colour indicates the strength of the effect of fruit size. We fitted a beta regression model for each species and extracted standardised coefficients for each fruit size. [file MEN-25-e70056-s001.zip › men70056-sup-0004-Supinfo4@Supplementary_20250908.docx]

Supplementary Materials for

**Airborne eDNA reveals resource-based assembly of frugivorous vertebrates**

**1. Regionally published researches (in Chinese) for checking the distribution of species:**

He RC, Wang L, Quan RC (2020). Introduction to Transboundary Animal Diversity Monitoring Platform of Southern Yunnan, China and Southeast Asia. *Biodiversity Science*, 28(9): 1097-1103. https://doi.org/[10.17520/biods.2020154](https://doi.org/10.17520/biods.2020154)

Pan QH, Wang YX, Yan K (2007). A Field Guide to the Mammals of China. *China Forestry Publishing House*.

Liu Y, Chen SH (2021). The CNG Field Guide to the Birds of China. *Hunan Science and Technology Press*.

Yan B, Wang LF, Liu SQ, Ji K, Mao YN, Zhang ZY (2020). Diversity of Animal Species on the Chinese Side of China-Laos Cross-border Biodiversity Joint Protection Area. *Forest Inventory and Planning*, 3, 42.

Zhang MX, Cao L, Quan RC, Xiao ZS, Yang XF, Zhang WF, Wang XZ, Deng XB (2014). Camera trap survey of animals in Xishuangbanna Forest Dynamics Plot, Yunnan. *Biodiversity Science*, 22, 830. <https://doi.org/10.3724/SP.J.1003.2014.14064>

**2. Figure S1:** Estimated site occupancy coefficients and 95% Bayesian credible intervals (95% CI) for (a) birds and (b) mammals. (a) Effect sizes of covariates on occupancy probabilities for birds; (b) effect sizes for mammals. Each bar in the graph shows the estimated effect of a covariate on site occupancy. Bars indicate statistically significant effects, where the 95% credible intervals do not overlap zero.

**3. Figure S2:** Estimated site occupancy coefficients and 95% Bayesian credible intervals (95% CI) for (a) Frugivores and (b) Carnivores (c) Omnivores: Each bar represents the effect size of a specific coefficient on site occupancy. The green bar indicates variables with statistically significant effects (credible intervals not overlapping zero). The number of species included in the analysis is indicated by the letter 'n'.

**4.** **Figure S3:** The influence of fruit size classes on the occupancy of single species. The colour indicates the strength of the effect of fruit size. We fitted a beta regression model for each species and extracted standardised coefficients for each fruit size.
