# Supplementary figures and images for: Airborne eDNA Reveals Resource‐Based Assembly of Frugivorous Vertebrates
Source: Mol Ecol Resour. 2025 Oct 10;25(8):e70056. doi: 10.1111/1755-0998.70056 (PMC12550463; doi:10.1111/1755-0998.70056)

Covariate

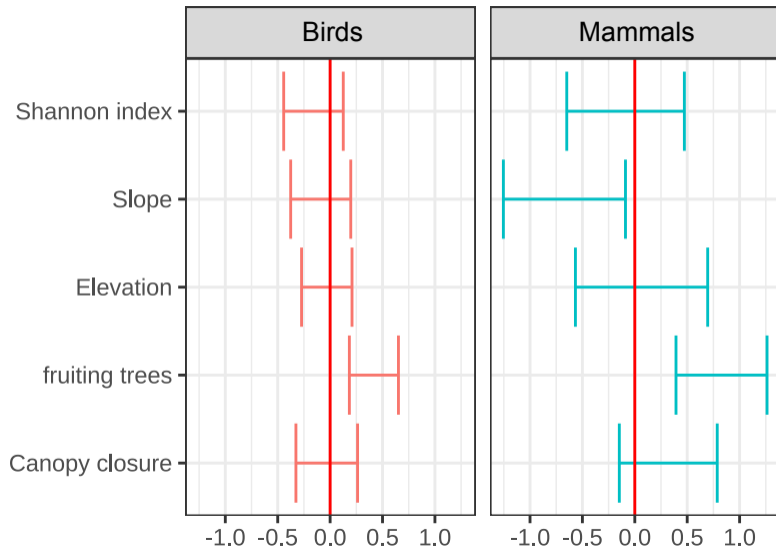

Supplement: Supplementary file 1 — Figure S1: Estimated site occupancy coefficients and 95% Bayesian credible intervals (95% CI) for (a) birds and (b) mammals. (a) Effect sizes of covariates on occupancy probabilities for birds; (b) effect sizes for mammals. Each bar in the graph shows the estimated effect of a covariate on site occupancy. Bars indicate statistically significant effects, where the 95% credible intervals do not overlap zero. Figure S2: Estimated site occupancy coefficients and 95% Bayesian credible intervals (95% CI) for (a) Frugivores and (b) Carnivores (c) Omnivores: Each bar represents the effect size of a specific coefficient on site occupancy. The green bar indicates variables with statistically significant effects (credible intervals not overlapping zero). The number of species included in the analysis is indicated by the letter ‘n’. Figure S3: The influence of fruit size classes on the occupancy of single species. The colour indicates the strength of the effect of fruit size. We fitted a beta regression model for each species and extracted standardised coefficients for each fruit size. [file MEN-25-e70056-s001.zip › men70056-sup-0001-Supinfo1@figs1.pdf]

**a**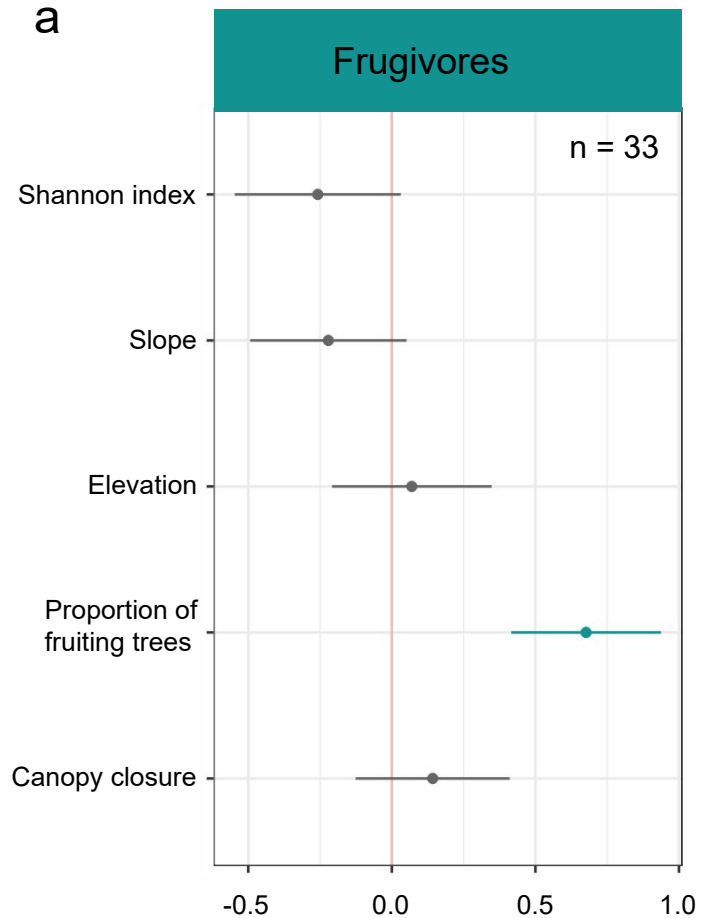**b**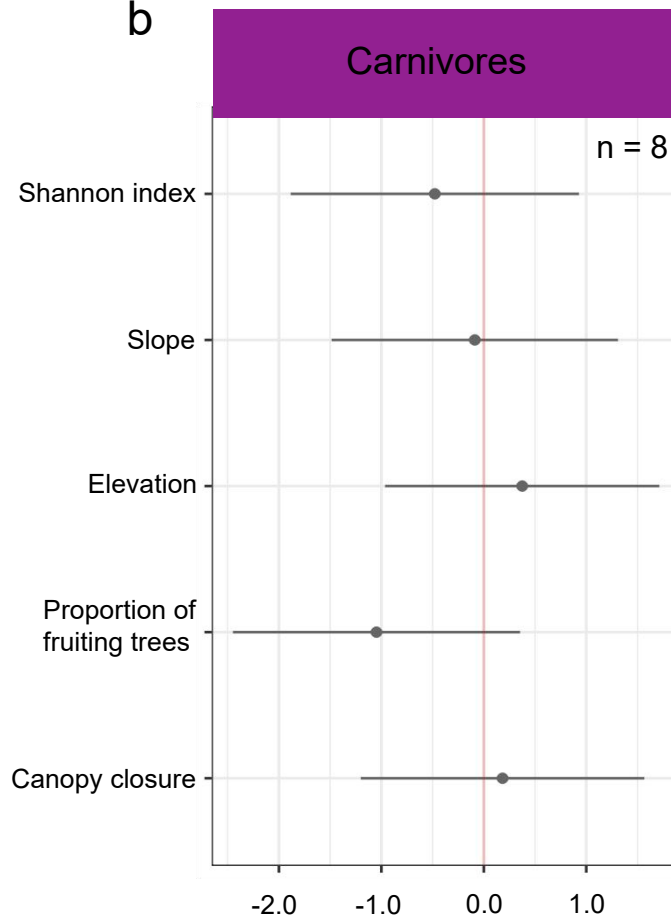**c**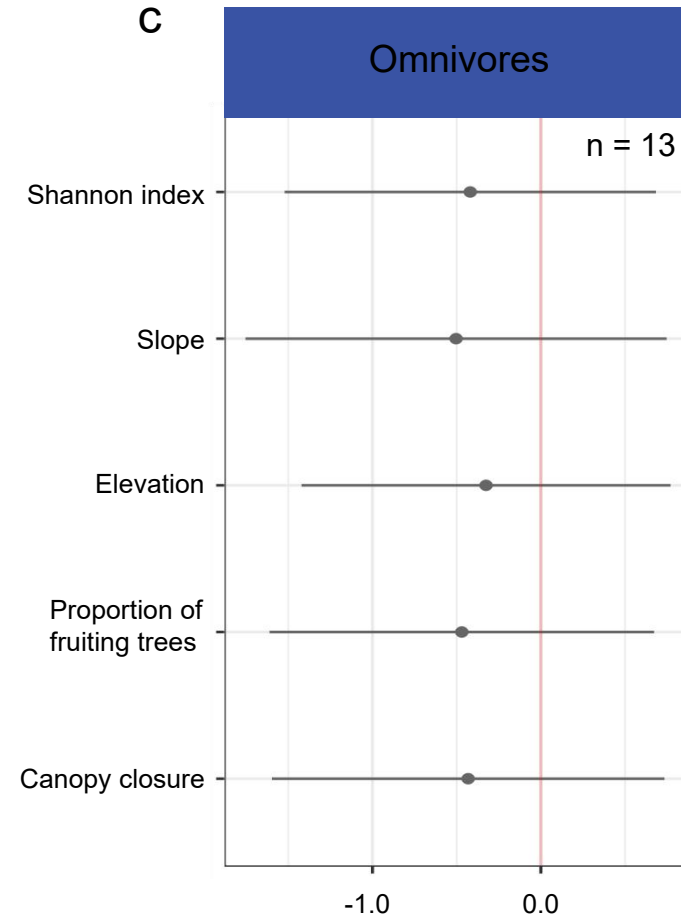

Supplement: Supplementary file 1 — Figure S1: Estimated site occupancy coefficients and 95% Bayesian credible intervals (95% CI) for (a) birds and (b) mammals. (a) Effect sizes of covariates on occupancy probabilities for birds; (b) effect sizes for mammals. Each bar in the graph shows the estimated effect of a covariate on site occupancy. Bars indicate statistically significant effects, where the 95% credible intervals do not overlap zero. Figure S2: Estimated site occupancy coefficients and 95% Bayesian credible intervals (95% CI) for (a) Frugivores and (b) Carnivores (c) Omnivores: Each bar represents the effect size of a specific coefficient on site occupancy. The green bar indicates variables with statistically significant effects (credible intervals not overlapping zero). The number of species included in the analysis is indicated by the letter ‘n’. Figure S3: The influence of fruit size classes on the occupancy of single species. The colour indicates the strength of the effect of fruit size. We fitted a beta regression model for each species and extracted standardised coefficients for each fruit size. [file MEN-25-e70056-s001.zip › men70056-sup-0002-Supinfo2@figs2.pdf]

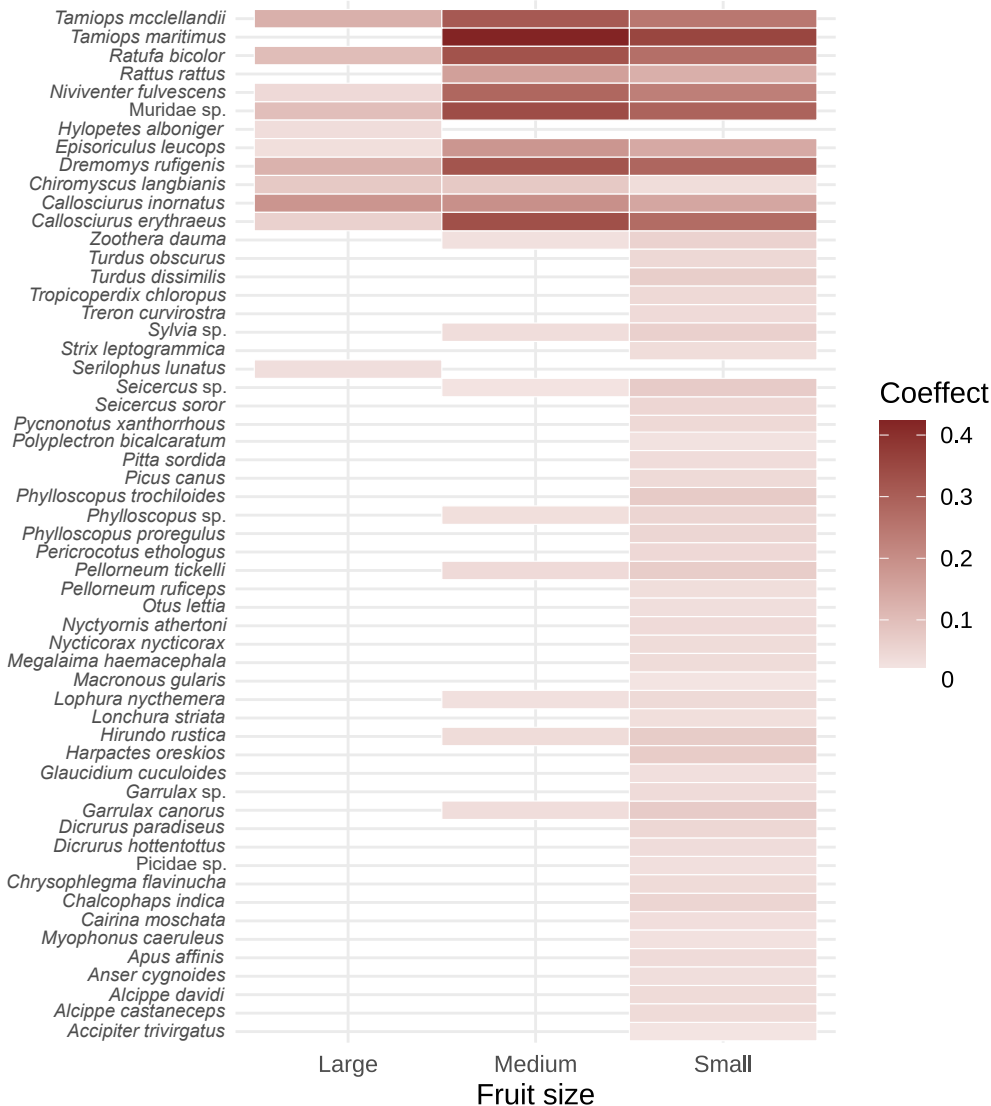

Supplement: Supplementary file 1 — Figure S1: Estimated site occupancy coefficients and 95% Bayesian credible intervals (95% CI) for (a) birds and (b) mammals. (a) Effect sizes of covariates on occupancy probabilities for birds; (b) effect sizes for mammals. Each bar in the graph shows the estimated effect of a covariate on site occupancy. Bars indicate statistically significant effects, where the 95% credible intervals do not overlap zero. Figure S2: Estimated site occupancy coefficients and 95% Bayesian credible intervals (95% CI) for (a) Frugivores and (b) Carnivores (c) Omnivores: Each bar represents the effect size of a specific coefficient on site occupancy. The green bar indicates variables with statistically significant effects (credible intervals not overlapping zero). The number of species included in the analysis is indicated by the letter ‘n’. Figure S3: The influence of fruit size classes on the occupancy of single species. The colour indicates the strength of the effect of fruit size. We fitted a beta regression model for each species and extracted standardised coefficients for each fruit size. [file MEN-25-e70056-s001.zip › men70056-sup-0003-Supinfo3@figs3.pdf]
